# Aortic Aneurysm and Dissection: Heterogeneity and Molecular Mechanisms

**DOI:** 10.3390/biom12101536

**Published:** 2022-10-21

**Authors:** Hong S. Lu, Hisashi Sawada, Congqing Wu

**Affiliations:** 1 Saha Cardiovascular Research Center and Saha Aortic Center, University of Kentucky, Lexington, KY 40536, USA; 2 Department of Physiology, University of Kentucky, Lexington, KY 40536, USA; 3 Department of Surgery, University of Kentucky, Lexington, KY 40536, USA; 4 Department of Microbiology, Immunology, and Molecular Genetics, University of Kentucky, Lexington, KY 40536, USA

Aortic aneurysms and dissections (AAD) are devastating aortic diseases with high risks for aortic rupture, leading to uncontrolled bleeding and death. Despite significant advances in our understanding of the disease pathogenesis, there are still many unanswered questions and conflicting findings requiring clarification. This Topical Collection of Biomolecules, aiming to highlight the pathological heterogeneity and molecular mechanisms of AAD, includes five reviews, three original research articles, two commentaries, and one perspective article.

Abdominal aortic aneurysms (AAAs) are the most common aortic aneurysms in humans. Several mouse models of AAAs were developed in the past two decades to study the disease’s development and related mechanisms. One common mouse model is infusing angiotensin II (AngII), an 8-amino acid peptide, into mice subcutaneously through mini-osmotic pumps [1]. It is worth noting that hypercholesterolemia drastically increases the incidence of AngII-induced AAAs in male mice [2,3]. However, to study AAAs by genetic manipulation in a target molecule, this mouse model requires breeding with either apolipoprotein E or LDL receptor −/− strain. This is both time- and cost-consuming [4]. Of note, adeno-associated viruses (AAVs) containing a gain-of-function mutation of PCSK9 can induce hypercholesterolemia in C57BL/6 mice within a week [5,6,7]. In a commentary of this Special Issue, Sawada and colleagues described the detailed method of how to use AAVs containing a mouse PCSK9 gain-of-function mutation [8]. In an original research article of this Special Issue, Ikezoe et al. [9] provided solid data that hypercholesterolemia by this PCSK9 AAV induction does not augment elastase-induced AAAs in mice, implicating potentially different mechanisms between AngII and elastase-induced AAAs.

Several articles of this Special Issue have provided comprehensive reviews on molecular mechanisms of AAAs. Thrombosis and aortic wall rupture are fatal consequences of AAAs, and platelet activation and aggregation have been reported by many investigators. Sun et al. [10] searched the PubMed and Science Direct database for “platelets and AAAs” and provided an extensive review and evaluation of the current knowledge on platelet activation as a mechanism and therapeutic strategy of AAAs. DeRoo et al. [11] reviewed the contributions of the endothelial layer, the frontmost barrier of the aortic wall, to the development of AAAs. In addition to summarizing the recent research findings using animal models and in vitro system, the authors [11] also reviewed the clinical evidence supporting effects and mechanisms of endothelial dysfunction in AAAs. Epigenetic modifications are important regulatory mechanisms of many diseases. Mangum and colleagues reviewed the present literature on epigenetic processing in the pathogenesis of AAA development [12]. Plasma serum amyloid A (SAA) is associated with multiple cardiovascular diseases. Shridas et al. reviewed the pathological significance of SAA in the development of atherosclerosis and AAAs [13].

The ultimate goal of our research is to treat AAAs. Fibrates are medications used to lower plasma triglycerides in patients with dyslipidemia. Amioka and Miyoshi [14] summarized the current literature that shows that fibrates have multiple beneficial effects on treating AAAs in mice, but the findings in human studies have not been consistent. The authors provided a comprehensive discussion on the discrepancy of the literature [14]. Edaravone is an FDA-approved antioxidant. Uchida and colleagues [15] administered this drug to male apolipoprotein-E-deficient mice infused with AngII. Edaravone did not affect plasma cholesterol concentrations and AngII-induced high blood pressure but reduced AAAs and atherosclerosis. The authors found that the attenuation of aortic pathologies was accompanied by diminished inflammation [15].

Thoracic aortic aneurysms (TAA) are the second most common aortic aneurysms. Asano and colleagues [16] provided an extensive review on TAAs attributed to a relatively common genetic disease in humans: Marfan syndrome. Many patients with Marfan syndrome suffer from TAA caused by genetic changes of an extracellular protein fibrillin-1. Mouse models with fibrillin-1 manipulations mimic the human disease. As reviewed by Asano et al., mouse models provided great mechanistic insights into TAAs of this devastating disease [16].

For aortic aneurysm research, irrespective of the location, it is important to apply appropriate and accurate imaging and quantification of aortic dilatation and pathologies in animal models. Ito and co-authors [17] reviewed the imaging techniques used for aortic visualization, discussed their advantages and limitations, and provided suggestions on how to choose the appropriate imaging technique.

This Special Issue also reports a new finding. Fludrocortisone is a drug that has been used in patients with orthostatic low blood pressure to increase blood pressure. Ye and co-authors [18] found that fludrocortisone induced aortic aneurysms in ascending, arch, descending thoracic, and suprarenal abdominal aortic regions. Different from AngII-induced aortic aneurysms, fludrocortisone-induced aortic pathologies occur in both normocholesterolemic and hypercholesterolemic mice. The presence of aortic aneurysms was not associated with blood pressure changes [18]. The findings in this article warn clinicians to closely monitor this possible fatal side effect when they prescribe fludrocortisone or drugs of the same class to patients.

We thank every author for their great contributions to this Special Issue. We hope that the articles published in this Special Issue will help researchers to improve our understandings of the pathological heterogeneity, molecular mechanisms, and potential therapeutic targets of AAD.
